# Understanding Environmental and Contextual Influences of Physical Activity During First-Year University: The Feasibility of Using Ecological Momentary Assessment in the MovingU Study

**DOI:** 10.2196/publichealth.7010

**Published:** 2017-05-31

**Authors:** Chloe Bedard, Sara King-Dowling, Madeline McDonald, Genevieve Dunton, John Cairney, Matthew Kwan

**Affiliations:** ^1^ Department of Health Research Methods, Evidence, and Impact McMaster University Hamilton, ON Canada; ^2^ INfant and Child Health (INCH) Lab, Department of Family Medicine McMaster University Hamilton, ON Canada; ^3^ Department of Kinesiology McMaster University Hamilton, ON Canada; ^4^ Institute for Health Promotion & Disease Prevention Research Department of Preventive Medicine University of Southern California Los Angeles, CA United States; ^5^ Faculty of Kinesiology and Physical Education University of Toronto Toronto, ON Canada

**Keywords:** exercise, compliance, feasibility studies, young adult, students

## Abstract

**Background:**

It is well established that drastic declines in physical activity (PA) occur during young adults’ transition into university; however, our understanding of contextual and environmental factors as it relates to young adults’ PA is limited.

**Objective:**

The purpose of our study was to examine the feasibility of using wrist-worn accelerometers and the use of ecological momentary assessment (EMA) to assess the context and momentary correlates of PA on multiple occasions each day during first-year university.

**Methods:**

First-year university students were asked to participate in the study. The participants completed a brief questionnaire and were subsequently asked to wear an ActiGraph GT9X-Link accelerometer and respond to a series of EMA prompts (7/day) via their phones for 5 consecutive days.

**Results:**

A total of 96 first-year university students with smartphones agreed to participate in the study (mean age 18.3 [SD 0.51]; n=45 females). Overall, there was good compliance for wearing the accelerometers, with 91% (78/86) of the participants having ≥2 days of ≥10 hours of wear time (mean=3.53 valid days). Students were generally active, averaging 10,895 steps/day (SD 3413) or 1123.23 activity counts/min (SD 356.10). Compliance to EMA prompts was less desirable, with 64% (55/86) of the participants having usable EMA data (responding to a minimum of ≥3 days of 3 prompts/day or ≥4 days of 2 prompts/day), and only 47% (26/55) of these participants were considered to have excellent EMA compliance (responding to ≥5 days of 4 prompts/day or ≥ 4 days of 5 prompts/day).

**Conclusions:**

This study represents one of the first studies to use an intensive real-time data capture strategy to examine time-varying correlates of PA among first-year university students. These data will aim to describe the physical and social contexts in which PA occurs and examine the relationships between momentary correlates of PA among the first-year university students. Overall, current results suggest that wrist-worn accelerometers and EMA are feasible methods for data collection among the young adult population; however, more work is needed to understand how to improve upon compliance to a real-time data capture method such as EMA.

## Introduction

### Background

There is mounting evidence that engagement in habitual moderate-to-vigorous aerobic physical activity (MVPA) provides many physiological and psychosocial benefits as well as attenuates the risk of over 25 chronic health conditions [1-3]. In Canada, it is recommended that adults over the age of 18 years engage in 150 minutes of MVPA per week [4]. Despite the known benefits of regular physical activity (PA), accelerometry data collected in the 2012-2013 Canadian Health Measures Survey suggest that approximately 20% of Canadian adults engage in the recommended levels of PA [5]. There is a documented widespread pandemic of physical inactivity across developed nations [6-10], and this is particularly salient among the emerging adult population (ie, ages 18 to 25 years) [11-16]. Specifically, the transition out of high school has been found to be a period of time marked by drastic declines in leisure-time PA participation [17-19]. These declines in PA during this life stage are particularly concerning because behavior patterns exhibited during emerging adulthood track through to adulthood [20]. Therefore, it is critical that we understand how to best attenuate these drastic declines in PA, thereby developing strategies to help facilitate PA behaviors during early adulthood.

Before effective interventions can be designed and applied, salient determinants of PA and the specific contexts in which PA occurs must be well understood. Current evidence suggests that self-efficacy, behavioral intentions, past behavior, time constraints, and changing academic pressures are important factors related to PA behaviors in young adults transitioning to university or college [14,21-25]. To date, few studies have investigated the environmental and contextual influences of PA as these young adults move away from home for the first time. For example, little is known about the specific times, places, and settings in which PA tends to take place, as well as how momentary changes in affective or mood states, state self-control, or PA motivations impact PA behaviors. Furthermore, extant literature is often limited due to cross-sectional or prospective designs with single, self-reported retrospective assessments of PA and its correlates [14,21-23]. The transition out of high school is considered to be the first major life transition that an individual faces during the life course, often resulting in corresponding changes in priorities and actions [26]. This reinforces the need for more research to understand how this transitory period influences PA cognitions and, potentially, its variations during this major transition period.

### Measurement Issues

Traditional methods examining salient determinants of PA use self-report surveys, requiring respondents to retrospectively report their perceptions, thoughts, and feelings related to general PA over a predetermined period of time (eg, in the last 2 months, over the past week). A major limitation of these self-reported measures is the presence of social desirability and recall biases [27,28]. Self-reported PA frequencies and durations have been correlated to social desirability and social approval personality traits, leading to an overestimation of activity levels [29]. Particularly within the PA literature, it has been noted that our ability to recall over the past week is limited and highly subject to over-reporting errors [30-32]. Even in an honest attempt to recollect PA behavior in the time between PA occurrence and the time at which the respondent is asked about it, many details become distorted, thus resulting in invalid answers reported on the survey [27,31]. Furthermore, psychosocial assessments will ask respondents to reflect back over time to generalize their thoughts, raising questions of ecological validity for these measures [33]. More recently, however, emerging literature is attempting to minimize the time between the events and reporting, using real-time data capture methods [34].

### New Measurement Tools

Ecological momentary assessments (EMA) have become increasingly popular as a real-time data capture method in PA research. Although the method itself is not entirely new, as there are many examples of researchers using diaries and logs to record momentary feeling states and behaviors [34,35], the proliferation of smartphones has created new opportunities to conduct EMA studies, while limiting the burden of constant recording of occurrences using the traditional paper-and-pencil method. Smartphones have become ubiquitous in the young adult population [36] and therefore represent an intuitive way to implement data-intensive recruitment strategies by collecting multiple responses from participants via electronic surveys sent multiple times throughout the day [34]. These data would also be advantageous as they represent more ecologically valid assessments that are representative of everyday life [34].

The emergence of objective PA measurement using devices such as accelerometers has also become increasingly popular due to their ability to capture the occurrence, duration, and intensity of PA [37]. More recently, validated wrist-worn accelerometers have become available for researchers, providing an alternative to the traditional waist-worn accelerometers, which can be more aesthetically appealing and comfortable to wear—both of which have been cited as reasons for noncompliance to waist-worn [38]. According to Troiano et al [39], the compliance rate for wrist-worn accelerometers tends to be higher than the compliance rate for waist-worn accelerometers, thus supporting the shift in research from waist-worn to wrist-worn accelerometers. In addition, results from the National Health and Nutrition Examination Survey (NHANES) study found that the compliance rate was 70% to 80% for participants providing valid data with wrist-worn accelerometers as compared with only 40% to 70% for those who provided valid data with waist-worn accelerometers [39]. Despite the emergence of these tools, few studies have investigated the utility of using these methods in PA research among young adults who recently transitioned out of high school.

### Study Purpose

The purpose of this study was to explore the feasibility of using wrist-worn accelerometers and using EMA as a data-capture tool to better understand the salient correlates of PA for young adults who recently transitioned from high school into university.

## Methods

### Participants and Recruitment

The sample used in this study consisted of 86 first-year university students living in university residences as a part of the MovingU study. The mean age of the sample was 18.31 years (SD 0.51), primarily white (38/85, 45%) or Asian students (26/85, 31%), with a relatively equal proportion of males (40/85, 47%) and females (45/85, 53%) (see Table 1). To be eligible, participants must have been in their first year of studies, living on campus, and have a smartphone capable of downloading the EMA app (iOS or Android operating systems). Participants were recruited over the course of a 2-week period at two university residence buildings. Recruitment materials (ie, social media and flyers) briefly outlined the study’s purpose and were advertised to all the students living in those residence buildings. All students interested in participating in the study were asked to meet with trained research assistants in a common area on a Tuesday evening.

### Procedures

All interested students were first provided with a detailed description of the study purposes and requirements. Participants who provided written consent were then asked to complete a Web-based questionnaire using an Android tablet. Once completed, participants were asked to download on their smartphone (iPhone or Android) the mobile EMA (mEMA) app designed and developed by illumavu Inc [40]. Each participant was given a unique personalized code to enter into the app, with EMA prompts scheduled to begin the following morning. A random sampling schedule was used, whereby participants were prompted at random times within predetermined time frames throughout the day (ie, every 2 hours) between 9:00 AM and 11:00 PM. Participants were instructed to complete all or as many of the EMA prompts over the 5-day sampling period and were also given a wrist-worn accelerometer (ActiGraph GT9X Link; Actigraph, Pensacola, FL, USA) to wear for each of those days. This 5-day sample is generally consistent with previous EMA research, which typically spans between 4 and 14 days [41]. With the exception of showering and participation in water-based activities, participants were instructed to wear the accelerometer for as long as possible (including sleep). Participants were provided a sleep log to indicate the times they went to bed each night and woke up each morning. They were also instructed to contact the study team if they encountered any issues with the EMA app. To compensate the students for their time and efforts, participants were given a Can $10 Starbucks gift card for completion of the questionnaire and agreeing to wear the accelerometer. For the additional burden of completing EMA prompts, they were compensated another $1 (in Starbucks gift cards) for each prompt they completed to a maximum of Can $5 per day (or Can $25 over the 5-day study period). All study procedures were approved by the Hamilton Integrated Research Ethics Board.

**Table 1:** Demographic characteristics of participants (N=85).

Characteristics		n (%) or mean (SD)
Age in years, mean (SD)^a^		18.3 (0.51)
**Gender, n (%)**		
	Male	40 (47)
	Female	45 (53)
**Ethnicity, n (%)**		
	White	38 (45)
	Asian	26 (30)
	South Asian	6 (7)
	Middle Eastern	5 (6)
	Black	3 (4)
	Indigenous	2 (2)
	Mixed race	3 (4)
	Prefer not to answer	2 (2)
12-point GPA^b^, mean (SD)		8.1 (2.3)

^a^SD: standard deviation.

^b^GPA: grade point average.

**Table 2:** Compliance rates for accelerometer wear time (N=86).

Category	Definition	n (%)
Excellent	≥10 hours of wear time for ≥5 days	20 (23)
Good	≥10 hours of wear time for 4 days	34 (40)
Fair	≥10 hours of wear time for 3 days	15 (17)
Minimum	≥10 hours of wear time for 2 days	9 (11)
Noncompliant	Did not have ≥10 hours of wear time for 2 days	8 (9)

### Feasibility Indicators

#### Accelerometer Compliance

Accelerometry is an objective method of assessing free-living PA and an established method for measuring activity [42]. Participants were instructed to wear the wrist-worn ActiGraph GT9X Link accelerometer on their nondominant wrist for 5 consecutive days. Given that the ActiGraph Link is relatively new, without validated cut-points relative to intensity of PA in this population, total counts of PA will be used and analyzed in 60s epochs. Compliance to accelerometer use was categorized into four domains distinguished by the number of days of valid wear time of 10 or more hours per day: minimal, fair, good, and excellent. The minimum compliance wear time of 10 hours per day is the most often used cut-off for accelerometry data in the United States and Canada [43,44]. Given that the purpose of this study was to describe the feasibility of using wrist-worn accelerometers, we categorized the data to reflect fair, good, and excellent compliance to accelerometer wear (see Table 2). Wear time was calculated using ActiLife based on the Troiano [43] wear time validation parameters with non-wear time defined as ≥60 minutes of consecutive zero counts.

#### EMA Compliance

Participants received 7 EMA prompts per day over the 5-day study period, resulting in 35 prompts in total. Using a random-sampling signal-contingent schedule, participants were asked to respond to prompts whenever a notification was sent to their device (taking approximately 1 to 2 minutes to complete). Consistent with several previous EMA studies [45,46], each prompt includes a very brief questionnaire, assessing contextual information on current activity, physical location, type of social company, current affective and feeling states, as well as state motivation to be active and self-control. Specifically, it included questions to obtain contextual information on three domains: “What are you currently doing?” “Where are you right now?” and “Who are you currently with?” Each question had a range of response options outlining expected responses and an “other” response option with a textbox for options not listed. Each prompt also included measures of acute outcome expectancy (eg, “doing 10+ min of physical activity in the next few hours would help me feel less stressed”), barrier self-efficacy (eg, “Can you do 10+ min of physical activity sometime within the next few hours even if you get busy?”), and intentions (eg, “I intend to be physically active for 10+ min sometime within the next few hours”), and was evaluated on a 5-point Likert scale. These items have been used in previous research and have been found to be valid measures [41,46-48]. Additionally, 5 items to assess affective states were included (eg, “How happy do you feel right now?” and “How tense or anxious do you feel right now?”), with response options ranging from “not at all” to “extremely,” which were based on the Positive and Negative Affect Schedule for Children [49]. Finally, we included 2 items from the State Self-Control Capacity Scale (eg, “If I were tempted by something right now, it would be very difficult to resist”), assessed on a 7-point Likert scale as used in Schöndube et al [50]. Further details on the MovingU study design, including the specific questions asked at each prompt, can be found in a previous publication [45]. Compliance to EMA prompts were categorized into 4 groups distinguished by the number of days prompts were answered and the number of prompts answered per day: noncompliant, minimum, fair, and excellent. The noncompliant to minimum compliance threshold identified was based on the minimum responses we estimate are required for using statistical techniques such as mixed-effects modeling (ie, at least 3 days of 3 prompts/day OR ≥ 4 days of 2 prompts/day). The categorizations for fair and excellent compliance were to further describe how compliant the participants were to the EMA prompts (see Table 3).

**Table 3:** Compliance rates for EMA responses (N=86).

Category	Definition	n (%)
Excellent	≥5 days of 4 prompts/day or ≥4 days of 5 prompts/day; response to ≥57% of total prompts.	26 (30)
Fair	≥4 days of 3 prompts/day or at least three days (including 1 weekend day) of 4 prompts/day; response to ≥34% of total prompts.	18 (21)
Minimum	≥3 days of 3 prompts/day or ≥4 days of 2 prompts/day; response to ≥23% of total prompts.	11 (13)	
Noncompliant	≤4 days of fewer than 2 prompts/day.	31 (36)

### Process Evaluation Survey

Following the 5-day study period, all participants were invited to voluntarily complete an anonymous Web-based questionnaire assessing the acceptability and receptivity of our EMA study. An invitation to participate was emailed to all participating students, including a link to the questionnaire on the Web. A total of 47 of the 86 study participants (55% response rate) completed the brief 10-item questionnaire related to *compliance*, *perceived burden*, and *compensation.*

**Compliance**

Three questions were asked related to participants’ general compliance to wearing the accelerometer or completing the EMA prompts. For example, we asked, “How often were you able to wear the accelerometer watch?” with responses ranging from 0=I didn’t wear it to 4=I wore it all the time; and “On average, how many prompts per day did you respond to?” with responses ranging from 0=none to 4=5 or more.

**Perceived Burden**

Two questions sought feedback regarding the perceived study burden, asking, “What did you think about the number of prompts that you received each day?” with the response options of 0=far too few, 1=somewhat too few, 2=just right, 3=somewhat too many, and 4=far too many; and “What did you think about the 5 days of being prompted?” with the response of 0=5 days was appropriate or 1=5 days was too long.

**Compensation**

There were 5 items that asked about compensation and participants’ overall motivation to participate in the study. For example, there was a question that asked, “What did you think of the study compensation or gift card amount?” Response options included 0=wasn’t worth my time, 1=too little for my time, 2=fair, 3=good for the time required, and 4= very good *,* generous for the time required.

### Analyses

Descriptive statistics were used to summarize the demographic characteristics of the study sample, compliance rates of both accelerometer wear and EMA prompt responses, as well as responses to the process evaluation survey. Statistical analyses were conducted using SPSS version 22.

## Results

### Sample Characteristics

A total of 98 students initially provided written consent to participate in the study. There were 2 students who provided formal withdrawal, 1 student due to smartphone incapability with the mEMA app and 1 student because of expressed participant burden. There were 10 students who expressed interest and provided written consent, but for one reason or another did not take part in any of the data collection activities (ie, survey, accelerometer, EMA responses). Details of the sample characteristics for all participants are shown in Table 1, with one exception. A single participant provided accelerometer data and responded to EMA prompts, but failed to complete the baseline survey. This participant was included in the compliance results but not in the demographic characteristic table. Sample characteristics of the 85 participants are presented in Table 1.

### Accelerometer Compliance

Of the 86 participants, a total of 91% (78/86) wore the activity monitor for at least two days for 10 or more hours on each those days (see Table 2). The majority of our sample (54/86, 63%) met the requirements for good or excellent compliance, meaning they had valid accelerometry data (ie, ≥10 hours of wear time) on at least 80% of the study period. Only 9% (8/86) of the sample was considered noncompliant, failing to wear the accelerometer for the minimum of 2 days for 10 or more hours.

### EMA Compliance

Of the 86 participants in our sample, only 55 (64%) provided what we estimated to be usable data for analyses (ie, ≥ 3 days of 3 prompts/day or ≥4 days of 2 prompts/day). Among those with usable data based on compliance, nearly half of the participants (26/55, 47%) had excellent EMA compliance (defined as having 5 days of 4 or more prompts each day or having 4 days of 5 or more prompts each day), approximately one-third (18/55, 33%) had fair compliance (defined as responding to 4 days of 3 or more prompts per day or at least 3 days including 1 weekend day of 4 or more prompts per day), and 20% (11/55) have only met the minimum compliance requirements. The average number of prompts that participants with minimum compliance responded to over the 5 days was 19.6 (19.6/35, 56%). A complete breakdown of compliance rates for EMA responses are shown in Table 3.

### Combined Accelerometer and EMA Compliance

Of the 78 participants with valid accelerometry data, 64% (50/78) met the minimum compliance threshold of EMA responses. There were 5 participants who had compliant EMA data but did not have compliant accelerometer data, and a total of 3 participants who were noncompliant for both EMA responses and accelerometer wear.

### Process Evaluation Survey

Among the 47 participants who completed the process evaluation survey, 91% (43/47) self-reported wearing the accelerometer most or all of the time and 89% (40/45; 2 participants skipped this question) reported answering 3 or more EMA prompts a day. In terms of the compensation, only 2% (1/47) reported that the gift card amount was either too little for the time required in the study or was not worth their time. However, at least 75% (35/47) reported that they would have responded to more EMA prompts if they were provided with more compensation for their time. The majority of participants, 51% (24/47) and 96% (45/47), reported that the number of prompts they received each day and the number of days of prompting, respectively, were appropriate. Only 4% (2/47) reported that 5 days of prompting was too long, but 34% (16/47) reported that there were either somewhat or far too many prompts.

## Discussion

### Principal Findings

Given the drastic declines in PA during the emerging adulthood period, it is becoming increasingly important to intervene before individuals form stable unhealthy behavioral patterns. Considering the ubiquitous nature of smartphones combined with the accessibility of accelerometry, there is a unique opportunity to collect precise estimates of PA and greater ecologically valid measurements of the salient PA influences to inform the design of interventions. This study demonstrated that it is feasible to combine methods of wrist-worn accelerometers and a mobile EMA app among first-year university students; however, more can be done to improve compliance.

The compliance rate for the accelerometers was good to excellent, with 91% of the sample providing valid accelerometry data. The proportion of our sample providing valid accelerometry data is comparable with that of waist-worn accelerometers used in the 2007-2009 Canadian Health Measures Survey. Additionally, the average number of steps per day, 10,895, is similar to the population average in Canada [51]. The similarity of both of our accelerometer compliance rate and average step count suggests that our sample was similar to the broader Canadian population [51].

The compliance rate for accelerometer wear, however, was considerably better than the compliance rate for EMA responses. Among the 86 participants in the study, only 55 responded for 3 or more days of 3 or more prompts per day or for 4 days of at least 2 prompts each day. This compliance rate somewhat aligns with the information obtained on the process evaluation survey, indicating that one-third of respondents thought there were too many prompts; however, critically, it is considerably lower than the compliance from a late adolescent sample from Southern California [52]. This difference in compliance rates may be in part due to differences in sample age, EMA sampling schedule, and compensation. In Dunton et al [52], the sample included high school students. As a result, the students may be more likely to appease research staff because they are not yet at the stage where they feel fully autonomous and independent, or they may be prompted by their parents to acquiesce with the research study requirements. Alternatively, there may be more novelty for high school students to participate in research, compared with the ambivalence that university students may be demonstrating as they often get asked to participate in a variety of studies on campus. This hypothesis is somewhat supported because 53% of our sample reported in the process evaluation survey that the “study was somewhat interesting, but (they) had other things to do,” suggesting that our sample was prioritizing other activities over participation in research.

Another difference between our study design and the Dunton et al [52] study was the prompting schedule. Participants in Dunton et al [52] were not prompted throughout school hours and, thus, were prompted less frequently overall. Given that more than one-third of the postevaluation respondents indicated that they received too many prompts, this may have resulted in the greater rates of noncompliance in this study. Future research aimed at investigating the specific factors predicting EMA responses is required. This could determine whether there may be specific times within a day or days within the study period that result in greater noncompliance.

Finally, the role of compensation may be something that can help to improve compliance rates. Although the vast majority of participants indicated their satisfaction with the compensation provided, over 75% reported that their compliance could be improved with increasing value of compensation per prompt. Interestingly, fewer respondents indicated they would improve their response rate if offered larger and guaranteed compensation regardless of the number of prompts they answered. The study by Dunton et al [52] compensated $100 for 14 days of responses to EMA, which was significantly higher than our maximum of $35 in gift cards that could be earned. While more compensation may be required more generally, future research may want to investigate the differences in compliance based on compensation per prompt or for a guaranteed compensation. The findings from our postevaluation survey would suggest that participants may have been more motivated to answer prompts based on the value attached to each prompt rather than the guarantee of a large gift card after study completion. The caveat, however, is that 95.7% of respondents indicated that the length of the study period was appropriate, yet the compliance was relatively modest. More research to help identify optimal compensation methods and study duration length is needed.

### Limitations

There are a number of limitations associated with this study. First, the issue of generalizability of the sample must be acknowledged. The sample in this study included a relatively small and homogeneous group of students living on campus, and relatively passive recruitment methods were used. In addition to a response bias, our findings may not be generalizable to the entire first-year university student population, particularly for those who do not live on campus. Second, as this study took place at a university, the lower compliance rate may be due to the students’ inability to answer prompts being given during class times. More research aimed at understanding factors related to overall compliance and compliance during certain times of the day is needed. Third, EMA compliance rates may be partly attributed to a lack of familiarity with the app, as the push notification that sent the EMA survey to the participants required participants to navigate into the app when prompted. This may not have been entirely intuitive for participants. Fourth, it may be possible that our single, brief 5-day data collection period is not representative of the student’s typical routine. It may have been a particularly busy week for students that may be impacting compliance, thus further studies using multiple EMA collection periods are needed. Finally, we acknowledge that the response rate for the process evaluation survey was low, with less than half the sample providing feedback related to the acceptability and receptivity of using EMA in university students. The low response rate may be in part due to the anonymity of the survey and no compensation being given to respond. Given that they were completed anonymously, individual responses cannot be matched to their objectively measured compliance rate, although the vast majority of respondents reported being largely compliant to both the accelerometer wear and EMA responses. This is indicative of a response bias, and warrants caution when interpreting the process evaluation results.

### Conclusions

Overall, the MovingU study represents one of the first studies to use an intensive data recruitment strategy through the use of EMA, aimed at understanding the factors related to PA during students’ transition from high school into university. Current results suggest that wrist-worn accelerometry is a feasible method to assess objective PA among the young adult population, but that more work is needed to understand how to increase compliance to real-time data capture methods such as EMA. A greater understanding of the predictors of compliance to the EMA and accelerometer protocol in a population of emerging adults will inform the design of the next large-scale EMA study. Future work will then explore some of the time-varying and time-invariant predictors of EMA compliance, as well as begin to examine how PA correlates measured multiple times each day across the 5-day study period relate to objectively assessed PA in these first-year university students. With enhanced knowledge regarding the salient predictors of PA in an emerging adult population, we can move toward designing interventions that target these predictors to have a larger impact on PA behavior change during this major life transition.

## Abbreviations

EMA: ecological momentary assessment
mEMA: mobile ecological momentary assessment
MVPA: moderate-to-vigorous aerobic physical activity
NHANES: National Health and Nutrition Examination Survey
PA: physical activity
